# Reference values of urine protein/creatinine ratio in healthy Dalian adults

**DOI:** 10.1002/jcla.24043

**Published:** 2021-10-07

**Authors:** Tong Liu, Bang‐Lu Xue, Bin Du, Tong Cui, Xin Gao, Yue‐Ying Wang, Bo Wang, Jin‐Long Wei

**Affiliations:** ^1^ Department of Laboratory Medicine Dalian Municipal Central Hospital Liaoning China

**Keywords:** adults, Reference value, Urine protein creatinine ratio (UPCR)

## Abstract

**Background:**

The urine protein/creatinine ratio (UPCR) is commonly used in current clinical practice. However, there are only few published clinical data on UPCR from large cohorts of Chinese adults. This study aimed to determine the overall and age‐ and sex‐specific UPCR reference values for healthy Dalian adults.

**Methods:**

According to the Clinical & Laboratory Standards Institute EP28‐A3c guidelines, 1321 healthy Dalian adults (646 men and 675 women) aged 20–69 years were enrolled. Urine protein and creatinine levels were analyzed in the random morning spot urine samples, and UPCR was calculated. The 95th percentile of the UPCR was used as the normal upper limit. The Mann–Whitney U test was used to test differences among groups.

**Results:**

The UPCR reference value was 141.7 mg/g for the entire cohort, 128.7 mg/g for men, and 150.8 mg/g for women. In addition, women had relatively higher UPCR values than men in the same age group. We also compared the UPCR reference values between different estimated glomerular filtration rate (eGFR) groups and found that women had significantly higher UPCR values than men in the normal eGFR groups.

**Conclusions:**

This study provides the overall and age‐ and sex‐specific UPCR reference values for healthy Dalian adults.

## Graphical abstract

The urine protein/creatinine ratio (UPCR) reference value was 141.7 mg/g for the entire cohort, 128.7 mg/g for men, and 150.8 mg/g for women. In addition, women had relatively higher UPCR values than men in the same age group.

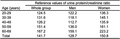

## 1 INTRODUCTION

Proteinuria is a well‐established independent risk factor for renal and cardiovascular diseases.^1^ Accurate measurement of proteinuria is of great importance in the diagnosis, disease activity monitoring, and prognosis of renal diseases.^2^ Although a 24‐h urine protein (24h‐UP) collection is regarded as the gold standard method for determining proteinuria, this test can be cumbersome and time‐consuming for patients and is sometimes subject to under‐collection. Based on the assumption that urinary creatinine and protein excretion are relatively constant throughout the day, Ginsberg et al. suggested a substitute method to avoid tedious urine specimen collections by detecting the urine protein/creatinine ratio (UPCR) in single random urine samples.^3^ Since then, several studies have shown a strong correlation between UPCR and 24h‐UP and have suggested that UPCR could be used instead of 24h‐UP in various diseases, such as rheumatic diseases, lupus nephritis, diabetes mellitus, and chronic kidney disease (CKD).^4^, ^5^, ^6^, ^7^ Interestingly, Ruggenenti et al. found that UPCR was more reliable than 24h‐UP measurement in chronic renal disease.^8^

The National Kidney Foundation practice guidelines recommend that UPCR be measured instead of 24h‐UP in most cases of children and adults with CKD, and the recommended reference value is lower than 200 mg/g.^9^, ^10^ Another widely used reference value is lower than 150 mg/g, which has been verified by many studies.^7^, ^11^, ^12^ However, these reference values were determined using data largely from Caucasian populations and did not take sex and age discrepancies into account. Therefore, we aimed to determine the UPCR reference values of healthy Dalian adults and establish their age‐ and sex‐specific reference values.

## 2 MATERIALS AND METHODS

### 2.1 Study population

Subjects who visited the health physical examination center of the Dalian Municipal Central Hospital between December 2019 and March 2021 were included in this study. They were screened by self‐reporting, specialized physicians' examinations, and clinical laboratory examinations. The inclusion criteria were as follows: (1) age 20–69 years and apparently healthy adults; (2) no self‐reported acute or chronic disease, pregnancy, and infections; (3) systolic blood pressure <140 mm Hg and diastolic blood pressure <90 mm Hg; (4) no hyperlipidemia, triglyceride <1.70 mmol/L, and total cholesterol <5.18 mmol/L; (5) negative dipstick testing for protein, glucose, blood cells, and white cells; and (6) biomarkers for coagulation, liver function, and renal function within the reference intervals used in our clinical laboratory. Enrolment of healthy individuals and data analysis followed the Clinical & Laboratory Standards Institute EP28‐A3c guidelines.^13^ A total of 1321 participants (646 men and 675 women) were included in the study.

### 2.2 Ethical statement

This study was approved by the Human Ethics Committee of Dalian Municipal Central Hospital and performed in accordance with the Declaration of Helsinki.

### 2.3 Urine protein and creatinine analysis

Random morning urine samples were collected after dipstick testing showed negative results for protein, glucose, blood cells, and white cells, stored at 4°C, and centrifuged at 212 *g* for 5 min. Total protein and creatinine levels in urine were measured within 2 h after collection using the colorimetric method (Randox Laboratories) enzyme colorimetric method on an ADVIA Chemistry XPT autoanalyzer (Siemens), respectively. To ensure that the results were reliable, low‐and high‐quality control of total protein and creatinine was conducted daily. We reviewed all available clinical and laboratory data on the subjects according to our inclusion criteria. We calculated the estimated glomerular filtration rate (eGFR) using the 2009 CKD‐Epidemiology Collaboration (EPI) equation.^14^

### 2.4 Statistical analysis

Skew distributional data are expressed as median and interquartile range. The 95th percentile was used to identify the normal upper limit of UPCR for all participants and for men and women in different age groups separately. For skew distributional variables, the Mann–Whitney U test was used to test differences among groups. Statistical analyses were performed using SPSS (version 23.0; IBM Corporation). All statistical analyses were performed using two‐sided tests, and statistical significance was set at *p* < 0.05.

## 3 RESULTS

Random morning urine samples were obtained from all eligible participants (N = 1321), including 646 men and 675 women. As shown in Table 1, there was no significant age difference between the sexes (*p* = 0.192), and men had higher serum creatinine (Scr), UP, and urine creatinine (Ucr) values but lower eGFR values than women (*p *< 0.0001).

**TABLE 1:** Characteristics of participants

	Total	Men	Women	*p* value
N	1321	646	675	
Age (year)	45 (32–57)	45 (32–56)	46 (33–57)	*p *= 0.192
eGFR (ml/min/1.73 m^2^)	109.4 (100.1–119.3)	107.7 (99.2–117.7)	111.5 (101.6–121.7)	*p *< 0.0001
Scr (μmol/L)	60 (51–71)	71 (65–76.5)	51 (47–56)	*p *< 0.0001
UP (mg/L)	59 (81–109)	85.5 (65–115.3)	74 (55–101)	*p *< 0.0001
Ucr (mg/dl)	136.5 (94.5–189)	157.7 (111.8–202.3)	115.9 (78.4–165.2)	*p *< 0.0001

Note

*p *< 0.05 was considered statistically significant.

Abbreviations: eGFR, estimated glomerular filtration rate; Scr, serum creatinine; Ucr, urine creatinine; UP, urine protein.

As shown in Table 2, the median and 95th percentile values of UPCR are presented by sex and age. According to the Mann–Whitney U test, women had significantly higher UPCR values than men (*p *< 0.0001). From the 40–49 to 60–69 age groups, increases in age were correlated with an increase in the median and 95th percentile UPCR.

**TABLE 2:** The median and 95th percentile of UPCR (mg/g) for men and women in different age groups

Age (years)	Total		Men		Women		*p* value
	Median	95th percentile	Median	95th percentile	Median	95th percentile	
20–29	52.4	124.5	49.5	122.2	58.6	136.3	*p *= 0.0002
30–39	55.5	131.6	53.6	118.1	58.0	145.1	*p *= 0.3893
40–49	57.0	126.2	54.2	112.7	61.1	135.9	*p *= 0.0226
50–59	64.0	151.4	59.1	136.0	65.3	161.1	*p *= 0.0297
60–69	69.9	167.2	65.4	159.1	74.9	223.2	*p *= 0.0263
Total	60.0	141.7	55.6	128.7	62.3	150.8	*p *< 0.0001

Note

*p *< 0.05 was considered statistically significant.

Similarly, the median and 95th percentile values of UPCR were presented by sex and eGFR (Table 3). A Mann–Whitney U test indicated that women had significantly higher UPCR values than men in the normal eGFR group (eGFR≥90 ml/min/1.73 m^2^). However, a higher eGFR value resulted in a lower median and 95th percentile of the UPCR.

**TABLE 3:** The median and 95th percentile of UPCR (mg/g) for men and women in different eGFR groups

eGFR (ml/min/1.73 m^2^)	Total		Men		Women		*p* value
	Median	95th percentile	Median	95th percentile	Median	95th percentile	
60–89	68.3	231.7	62.0	213.3	71.1	294.1	*p *= 0.8055
90–119	61.3	142.6	56.9	129.4	65.4	155.0	*p *< 0.0001
>120	57.8	136.1	51.7	105.8	60.0	149.1	*p *= 0.0062

Note

*p *< 0.05 was considered statistically significant.

## 4 DISCUSSION

Healthy individuals normally excrete very small amounts of protein in their urine; persistently high protein excretion is an indication of kidney damage.^9^ Accurate assessment of pathological proteinuria is important in the diagnosis, disease activity monitoring, and prognosis of patients with CKD.^15^ Owing to its simplicity and convenience, UPCR is widely used to evaluate daily protein excretion in current clinical practice, and the Kidney Disease Outcomes Quality Initiative of the National Kidney Foundation guidelines indicate that UPCR can be used instead of 24h‐UP for estimating proteinuria.^9^, ^10^ However, there are few published clinical data on UPCR from large cohorts of Chinese adults. In this study, we investigated the UPCR values in different age groups between men and women using random urine samples and established age‐ and sex‐specific reference values in healthy Dalian adults.

As the UPCR values had skewed distribution, we used the upper 95th percentile as the normal reference value. The reference value for the whole cohort was 141.7 mg/g which is obviously lower than that recommended by the National Kidney Foundation guidelines (200 mg/g),^9^ and slightly lower than the commonly used value (150 mg/g) according to previous studies.^3^, ^7^, ^11^, ^12^, ^16^ This discrepancy may be explained based on some aspects. First, the detection method and equipment for UP and Ucr used here have inevitable differences with those used in previous studies. Second, because the reported UPCR reference values were determined using data mainly obtained from Caucasian subjects, the racial differences in dietary protein intake, muscular mass, and physical exercise may result in the discrepancy.^17^ Finally, the relatively stricter inclusion criteria used in our study may partly account for this discrepancy.

Similar to the findings of the study by James et al., we found that men excreted more creatinine than women,^18^ which may explain why the UPCR value for women (150.8 mg/g) is higher than that for men (128.7 mg/g). In addition, it was revealed that with increasing age, there was a corresponding increase in the median UPCR value but not in the 95th percentile. Interestingly, Xu et al. showed similar results, but they mainly studied urine albumin to creatinine ratio in healthy Chinese adults.^19^ We also observed that from the 40–49 to 60–69 age groups, an increase in age was related to an increase in the median and 95th percentile UPCR. The relative decrease in skeletal muscle mass and kidney function may partly account for this phenomenon.^20^, ^21^, ^22^ Therefore, it is necessary to establish different reference ranges for UPCR for different age groups.

At present, GFR is considered the best measure of overall kidney function.^10^ The eGFR was calculated using the 2009 CKD‐EPI equation. Similar to the findings of Kosmadakis et al,^23^ we found that a high eGFR corresponded to a lower median and 95th percentile of UPCR. In addition, we found that women had significantly higher UPCR than men did in the normal eGFR group (eGFR≥90 ml/min/1.73 m^2^). However, it should be recognized that Scr and Ucr are present in the formulae used to estimate GFR and UPCR, respectively. Therefore, the UPCR reference values in different kidney functions should be used cautiously among subjects with abnormal creatinine generation and excretion.

However, our study has some limitations. First, we detected UPCR only in one random morning urine sample for each subject, which may lead to a certain degree of variation. Second, since the subjects included in our study were apparently healthy adults and the dipstick was negative for protein in all samples, the correlation between 24h‐UP and UPCR was not calculated. Finally, this study was a single‐center, hospital‐based study; therefore, unmeasured bias may exist. Despite these, this study also has advantages. This study is the first to investigate the UPCR reference values in a relatively large population of healthy Chinese adults aged 20–69 years. All patients underwent biochemical tests performed in a single laboratory.

In conclusion, we determined the UPCR reference values and established age‐ and sex‐specific reference values in healthy Dalian adults. Furthermore, we determined the UPCR reference values for different eGFR groups. Future studies are required to determine the effectiveness of these UPCR reference values in screening for proteinuria to prevent CKD.

## CONFLICT OF INTEREST

The authors report that they have no conflict of interest.

## Data Availability

All data included in this study are available upon request by contact with the corresponding author.
